# Supplementary material for: Phytomediated stress modulates antioxidant status, induces overexpression of CYP6M2, Hsp70, α-esterase, and suppresses the ABC transporter in Anopheles gambiae (sensu stricto) exposed to Ocimum tenuiflorum extracts
Source: PLoS One. 2024 May 2;19(5):e0302677. doi: 10.1371/journal.pone.0302677 (PMC11065307; doi:10.1371/journal.pone.0302677)
Supplement: Attachment — Submitted filename: PONE-D-24-03688.docx [file pone.0302677.s006.docx]

The authors investigate the larvicidal effects and putative molecular mechanisms of Ocimum tenuiflorum extracts against the malaria vector Anopheles gambiae. This research is driven by the need for environmentally-safe mosquito control alternatives to replace synthetic insecticides. The topic is relevant and the paper is within the scope of the journal PLOS One. Overall, the manuscript is clearly written and the research approach is sound. However, there are a few areas that need strengthening:

1- The introduction could provide more background on the specific phytocompounds found in O. tenuiflorum that have known bioactivity and relate this to the rationale for testing this plant.

2- The sample size used in larval bioassays is quite small at n=20. Increasing to at least n=30 per treatment would give more statistical power to detect differences between doses and extract types.

3- The toxicity mechanisms focus only on gene expression changes. Incorporating measurements of enzyme activity would significantly strengthen the link between transcript and protein induction.

4- Expanding the transcriptomic analysis to include other detoxification and stress response genes could provide greater coverage of the molecular pathways involved. ABC transporters alone give limited mechanistic insight.

5- Assessing chronic toxicity over the full larval development would better reveal sub-lethal impacts compared to acute toxicity alone.

This paper presents a worthwhile study on plant-based mosquito control, but lacks sufficient depth in some areas. Addressing the above points would significantly improve the quality of the results and strengthen the conclusions that could be drawn from this research. With moderate revisions to expand the scope, this work would represent a solid contribution to the field.
