# Supplementary material for: Phytomediated stress modulates antioxidant status, induces overexpression of CYP6M2, Hsp70, α-esterase, and suppresses the ABC transporter in Anopheles gambiae (sensu stricto) exposed to Ocimum tenuiflorum extracts
Source: PLoS One. 2024 May 2;19(5):e0302677. doi: 10.1371/journal.pone.0302677 (PMC11065307; doi:10.1371/journal.pone.0302677)
Supplement: Attachment — Submitted filename: Response to the Reviewers.docx [file pone.0302677.s008.docx]

**Response to the Reviewers’ comments**

Manuscript Number: PONE-D-24-03688R1

Article Title: Phytomediated stress modulates antioxidant status, induces overexpression of CYP6M2, Hsp70, α-esterase, and suppresses the ABC transporter in *Anopheles gambiae* (sensu stricto) exposed to *Ocimum tenuiflorum* extracts

Journal Title: PLOS ONE

The authors express their gratitude to the editor and thank the anonymous reviewers for their thorough and valuable review, suggestions, and comments on how to improve the paper's quality and accurate reporting of data. We have taken into account all of the feedback we have received in the amended manuscript.

All corrections are highlighted in red

**Academic editor’s comments**

Comment 1: Please review your reference list to ensure that it is complete and correct. If you have cited papers that have been retracted, please include the rationale for doing so in the manuscript text, or remove these references and replace them with relevant current references. Any changes to the reference list should be mentioned in the rebuttal letter that accompanies your revised manuscript. If you need to cite a retracted article, indicate the article’s retracted status in the References list and also include a citation and full reference for the retraction notice.

**Response**

- Thank you for this feedback and comment. We have made the following corrections to the references;

i. The citation, Kamaraj and Rahuman was removed because it was not part of the reference list and replaced with the accurate citation of Ragavendran [16]. (Line 317).
ii. Reference [39] which is the first citation in the discussion was changed to reference [24] in order to follow the chronological order of referencing as stipulated in the authors guideline. Therefore, this change affected the numbering of citations between 24 – 39. Consequently, previously citations of [24], [25], [26], [27], [28], [29], [30], [31], [32], [33], [34], [35], [36], [37], [38] and [39] were changed to [25], [26], [27], [28], [29], [30], [31], [32], [33], [34], [35], [36], [37], [38], [39], [40] respectively. These corrections were also equally done in the Reference list.

- Also, we have checked the references list and none of the cited papers have been retracted.

**Reviewer #1**

Comment 1: The manuscript requires language revision.

**Response**

- We have subjected the manuscript to Grammarly checker to further present the sentences with better clarity and coherence.
